# Environmental DNA monitoring of waterfowl reveals community changes during migration

**DOI:** 10.1371/journal.pone.0337508

**Published:** 2026-04-28

**Authors:** Luciana Guimaraes de Andrade, Steve Michael Bogdanowicz, Holger Klinck, David Lodge, Jose A. Andrés

**Affiliations:** 1 Department of Ecology and Evolutionary Biology, Cornell University, Ithaca, New York, United States of America; 2 K. Lisa Yang Center for Conservation Bioacoustics, Cornell Lab of Ornithology, Cornell University, Ithaca, New York, United States of America; 3 Cornell Atkinson Center for Sustainability, Cornell University, Ithaca, New York, United States of America; UTRGV: The University of Texas Rio Grande Valley, UNITED STATES OF AMERICA

## Abstract

Large-scale monitoring of wildlife populations is a cornerstone of effective conservation and resource management, particularly for highly mobile taxa whose distributions shift rapidly across space and time. In this paper we explore the possibility of using environmental DNA-based surveys as a cost-efficient, complementary tool to estimate populations of North American waterfowl species. To achieve this, we first evaluated the performance of all currently available avian metabarcoding primers and compared them to newly designed primers targeting the mitochondrial ND2 gene within the Anatidae tribes of North America. All the existing avian assays showed strong cross-priming amplification with other vertebrates. In contrast, in-silico analyses of our waterfowl targeted assays showed a high degree (>90%) of avian specificity, encompassing all the 132 Anatidae species sequenced thus far. We used this targeted metabarcoding approach to track the temporal variation in the relative abundance of waterfowl species during the fall migration at Montezuma National Wildlife Refuge, New York, a major resting area for waterfowl on their journey to and from North American nesting areas. We compared eDNA results with visual surveys conducted by us and from those reported on eBird, a community science database. Our results showed that eDNA detected all waterfowl species (n = 25) observed during the visual surveys. Positive correlations existed between standardized amplicon sequence variant (ASV) counts and the relative abundance of waterfowl species as reported in eBird on the day of sampling and up to five days prior. Importantly, eDNA metabarcoding captured temporal shifts in community composition and species turnover during fall migration, highlighting its utility for tracking relative changes in waterfowl assemblages through time. As is often reported in metabarcoding studies, eDNA did not provide a good metric for absolute abundance of species; accordingly, only 8 out of 25 waterfowl species showed significant correlations between the number of eDNA reads and the total abundance of birds. Overall, while eDNA-targeted metabarcoding has not yet been applied to study bird communities extensively, our results demonstrate that this technique can be used as an effective complementary tool for assessing species composition of waterfowl communities and estimating relative abundance of species within those communities.

## Introduction

Large-scale wildlife monitoring programs play a crucial role in meeting the management and conservation objectives for waterfowl (Anatidae), a taxonomic group that is ecologically, economically, and culturally significant [1]. To estimate population sizes and trends, traditional waterfowl monitoring efforts cover extensive geographic areas using a combination of visual surveillance techniques [2–9]. Ground visual surveys are labor-intensive and constrained by limited access to private lands [10]. Thus, aerial surveys have become the preferred tool in these large-scale surveys (e.g., [11]).

Despite their advantages, aerial surveys also have important limitations beyond the disturbance associated with low-altitude flights [12]. Depending on the sampling design, extrapolation to unsampled areas may be difficult and spatial or temporal shifts in waterfowl distributions may lead to underrepresentation of areas with high use [6]. Additionally, the infrequent timing of many aerial surveys constrains their ability to capture short-term variation in abundance, and the processing and interpretation analysis of aerial imagery remains time-consuming even with recent advances in machine-learning-based approaches [5,13–15].

Considering these constrains, there is a growing need for alternative, cost-effective, and minimally invasive tools for monitoring waterfowl populations. In this context, high-throughput sequencing of macro-organismal DNA from environmental samples, known as eDNA metabarcoding, has proven to be a robust and efficient technique to characterize biological communities across a diverse array of aquatic taxa (e.g., [16–20]. Several lines of evidence suggest that extending eDNA monitoring to waterfowl populations should be feasible.

First, eDNA metabarcoding surveys using fish and mammal primers typically “bycatch” bird species [21–28]. Second, analyses of water samples from zoo bird enclosures and natural environments have demonstrated that avian metabarcoding primers can be used to describe bird communities [29,30]. Third, eDNA assays have detected geese and swan species in both controlled and natural aquatic systems [31,32]. In this study we take a first step towards the future implementation of robust eDNA-based waterfowl biosurveillance programs by addressing two questions. Because waterfowl communities are highly dynamic, with rapid turnover during migration, effective monitoring must resolve temporal changes in community composition rather than relying solely on static presence or abundance. Accordingly, we test whether (1) a diverse waterfowl community can be reliably detected from water samples, and (2) whether eDNA-derived patterns of waterfowl abundance and turnover are consistent with those obtained from intensive ground-based visual surveys.

To answer these questions we first tested a series of published bird metabarcoding primers [30,33–39]. Although most of these primers successfully amplified waterfowl eDNA, our initial results showed PCR primer bias and cross-amplification with other abundant taxa, including fish, severely affected the sensitivity of the assay. To minimize these limitations we designed a metabarcoding assay [*sensu* [40]] specifically to detect North American waterfowl species. We then examined the applicability of this method by tracking the temporal variation in relative abundance of waterfowl species during fall migration at Montezuma National Wildlife Refuge, New York, a major resting area for waterfowl on their migration to and from North American nesting areas.

## Materials and methods

### Study areas

**Fig 1:**
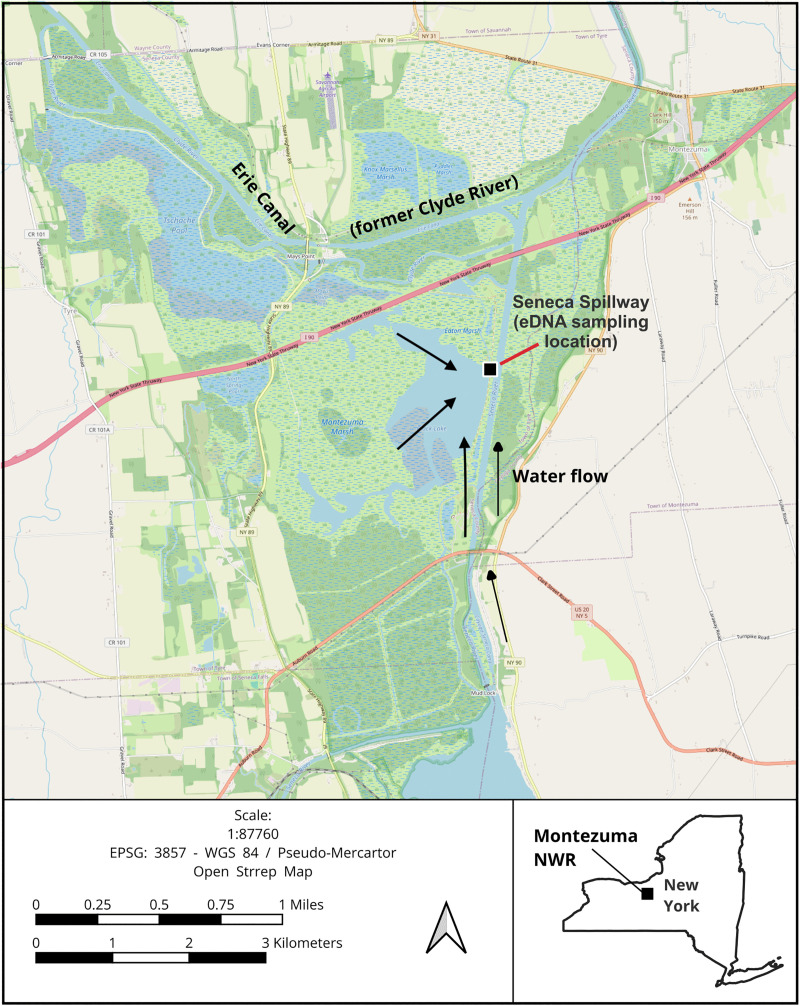
Water flow in the Main Pool at Montezuma NWR. The map shows the Seneca Spillway where water samples were taken in 10 consecutive weeks during the peak of the 2020 waterfowl fall migration. Black arrows represent the general direction of the water flow during this period.

Our primary study area was Montezuma National Wildlife Refuge, in Seneca County, New York, United States (Fig 1). Given the water flow within the aquatic system, our water samples primarily represented the Main Pool (Fig 1), but would also have contained water deriving from other parts of the system.

Montezuma NWR is a 4,000-hectare wetland complex located on a major migratory corridor in the Atlantic Flyway, sustaining up to a million waterbirds during non-breeding seasons [41]. The Main Pool (also referred to as Black Lake on some maps) is a shallow 309-hectare impoundment whose water levels are managed to provide emergent marsh and other habitat types for migratory birds and resident wildlife. The water level was 1 m deep during our field sampling, from September to November 2020. Other than precipitation, the primary intake for the Main Pool is water from Cayuga Lake (to the south), which is provided through a ditch with a gravity-fed connector [42]. Water leaves the Main Pool via the Seneca Spillway outlet, which empties into the Cayuga-Seneca Canal. The overall flow of water is from Cayuga Lake northward through the Main Pool to the Cayuga-Seneca Canal (Fig 1). To conduct the initial assessment of both general and targeted bird metabarcoding primers, we collected water samples from both Sapsucker Woods Pond in Tompkins County, New York (42°28’48.1"N, 76°27’05.7"W) and two water bodies at the Huyck Preserve (Lincoln Pond – 42°31"35"N, 74°9’31"W and Lake Myosotis – 42°31’ 38.3"N, 74°09’30.4"W) in Albany County, New York (S1, S2 and S3 Figs). Then, to test the validity of targeted metabarcoding as a monitoring tool, we took weekly water samples (n = 3 + 1 field blank) at Montezuma NWR during the peak of the 2020 fall waterfowl migration (September 24 -November 29). All weekly water samples were taken near the Seneca Spillway outlet (Fig 1). Sampling was carried out in compliance with the Montezuma National Wildlife Refuge Research and Monitoring Special Use Permit #20−05 issued by the U.S. Fish and Wildlife Service. Sampling at the Huyck Preserve was conducted in compliance with the funding received from the station.

### Water sampling

At each sampling location, we sampled eDNA by collecting and filtering 250 ml water samples and one distilled water negative control. This sample volume is commonly used in aquatic eDNA protocols and metabarcoding studies, providing a practical balance between capturing sufficient DNA and limiting filter clogging and field processing constraints (e.g., [43]).

Water was collected by submerging a sterilized wide-mouth Nalgene container (Thermo Fisher Scientific) < 0.1 m beneath the water’s surface following the recommendations of [44]. Each of the samples was then filtered through a 25 mm diameter cellulose-nitrate filter, 1.0 µm pore size, (Whatman, GE Healthcare) using a luer-lok sterile syringe system. Filtration blanks of distilled water were processed in the same way. Filters from each sample were immersed in 700 μl Longmire’s buffer in the field, transferred to the lab within 24 h, and stored at −80°C until DNA extraction. Water samples described above were taken just after our visual surveys.

### Visual surveys

From the shore, we conducted exhaustive visual surveys of all waterfowl on the Montezuma NWR Main Pool during weekly visits from September 24 to November 29, 2020. These ground-based surveys were performed along a 1.3 km stretch of the Montezuma NWR Wildlife Drive, immediately adjacent to the Main Pool, using Zeiss 10 x 40 binoculars and a Swarovski spotting scope with a 20–60x eyepiece. The average survey start time was 9:28 am (range = 7:47 am to 10:33 am) and the average survey duration was 131 minutes (range = 92–149 minutes). To supplement our ground visual surveys, we compiled waterfowl observational data from eBird, the world’s largest curated database of citizen science bird sightings [45], for each water sampling date plus a five-day look-back period. We followed best practices for using eBird data [46], utilizing only complete checklists (i.e., all the birds observed are reported) that followed eBird’s “Stationary” or “Traveling” protocols. For dates with more than one complete eBird checklist, we calculated species abundance as the mean number of individuals from all complete checklists.

### Avian metabarcoding primer tests using natural eDNA

Based on previous bird eDNA and phylogenetics studies we tested 11 unique primer pairs targeting different fragments of 5 mitochondrial loci: cytochrome c oxidase I [35,37], 12 S rRNA [30,34], NADH dehydrogenase subunit 2 [38,39], and Cytochrome b [33,36] (S1 Table). Insert length and amplicon size for each genetic marker were presented in S2 Table. The performance of each primer pair was tested on the eDNA samples from Sapsucker Woods Pond and Huyck Preserve (see above).

### Anatidae primer design

We chose the mitochondrial gene NADH Dehydrogenase, ND2, as the molecular marker for waterfowl species because it is a well-established locus in avian genetics that provides high taxonomic resolution among closely related bird species and has been widely used in phylogenetic and species-level identification studies, often outperforming more conserved mitochondrial loci at shallow evolutionary scales (e.g., [47]). In addition, ND2 is represented by an extensive and well-curated set of avian reference sequences in the GenBank database, facilitating reliable taxonomic assignment in metabarcoding applications when appropriate quality controls are applied.

All ND2 gene sequences from NCBI corresponding to all North American Anatidae tribes as of May 27, 2020 were downloaded, and up to two DNA sequences for each species from distant geographic locations were selected. A total of 85 sequences representing 54 species and 19 genera in the family Anatidae were then aligned using the ClustalW algorithm. The manual inspection of the alignment did not reveal suitable ND2 regions for a single “universal” set of primers for Anatidae. Thus, we followed a strategy similar to that of [48] to generate a Tamura-Nei NJ tree to identify groups of similar sequences sharing relatively conserved regions (S5 Fig). For each of the three groups we found, we designed a single set of PCR primers using the software Geneious Prime version 2020.1.1 and default parameters (Table 1).

**Table 1:** ND2 primers developed for DNA amplification of waterfowl species. Insert length, amplicon size and primer sequence for each waterfowl primer were included.

Primer	Insert length	Amplicon size	Targeted group	Direction	Primer sequence (5’ - 3’)
197F	297	332	Anserini/Cygnini	Forward	ACTTCCTRACCCAAGCAG
524R	297	332	Anserini/Cygnini	Reverse	ATTCAGCCYCCYAGYGC
419F	200	241	Anatini/Cairinini/Oxyura	Forward	TYATRAARTTYCCCCCRC
659R	200	241	Anatini/Cairinini/Oxyura	Reverse	GATGTYATGATYGYRTARAGRTA
220F	179	217	Aythyini/Mergini	Forward	TCCGCYYTWGTCCTRTTCT
436R	179	217	Aythyini/Mergini	Reverse	GTGGRGGRAAYTTYATGAG

### In-silico assessment of Anatidae ND2 primers

The performance of the primers was tested as follows. First, we used CRABS [49] to generate a custom reference database including all ND2 vertebrate sequences found in the NCBI nr repository (n = 157,730). Using the same pipeline, we performed in-silico PCR to produce a list of unique metabarcodes and assessed the specificity of the primers by calculating the percentage of Anatidae species amplified over non-target organisms using a maximum mismatch of 2 nucleotides in each primer.

### DNA extraction and library construction

All DNA extractions were carried out in a dedicated eDNA laboratory at Cornell University (Ithaca, NY), with UV light treatment overnight, HEPA filtered air under positive pressure, personnel full body suits, breathing masks and face cover shields. We used the DNeasy Blood and Tissue extraction kits (Qiagen Inc.) and the protocol described in [50]. All lab reusable materials, such as forceps, containers, and the lab counters, were regularly sterilized with 50% bleach solution and treated under UV light before and after each round of DNA extractions. Extraction negative controls were included in each of the eDNA extractions rounds. Extracted samples were placed in a −20^º^C freezer until library preparation. First-stage eDNA amplification was conducted in triplicate for each sample and negative control (i.e., field, filtration, extraction, and PCR blanks) in PCR volumes of 20 μl containing 8.475 μl molecular biology grade (MBG) water, 5 μl of 5x OneTaq Standard Reaction buffer, 0.2 μl of 10 μM primer (forward and reverse), 0.5 μl of 10mM dNTPs, 0.125 μl of One Taq Hot Start polymerase, 2.5 μl of bovine serum albumin (0.4 μg/μl), and 3 μl of eDNA extract as template. PCR cycling for each waterfowl ND2 primer pair was described in the supplementary material.

PCR products were then pooled by sample and tagged in a second-stage PCR using Illumina Nextera N7 and N5 indexes. The second PCR was carried out with a 20-mL reaction volume per eDNA sample containing: 3 μl DNA, diluted with MGB water 1:1, 10.9 μl MGB water, 4 μl 5x OneTaq buffer, 0.4 μl 10mM dNTPs, 0.8 μl each of 10 mM Nextera N7 and Nextera N5, and 0.1 μl OneTaq Hot Start DNA polymerase (New England – BioLabs Inc.).

PCR was run using the following thermal cycling parameters: six cycles of 30 seconds denaturation at 95ºC, 1 minute annealing at 62ºC, and 1 minute extension at 68ºC, with a final extension for 10 minutes at 68ºC. The resulting amplicons were purified and size-selected using Agencourt AMPure XP beads (reaction ratio AMPure beads 0.9: PCR product 1; Beckman Coulter Genomics), and the concentration of each library was estimated using the Qubit dsDNA Broad Range Kit and Qubit 2.0 fluorometer (Life Technologies). We pooled the barcoded PCR products for Anatini and Aythyini primers and all the libraries were diluted to 2 nM for paired-end sequenced V2 (2 x250bp) on a Miseq platform (Illumina, Inc.).

### Bioinformatic analyses

Initial quality control of raw sequence data was performed using FastQC/MultiQC [51]. Degenerate bases and adaptors were trimmed from demultiplexed paired reads and sequences 35 bp in length were removed using Trimmomatic 0.33 [52]. Processed sequences were then analyzed using DADA2 1.16 [53], which involved removing forward and reverse primers, trimming reads to 200 bp for Anatini/Aythyini primers, and 390 bp for Anserini/Cygnini, and discarding sequences with expected number of errors >2 (EE, calculated based on Phred scores). The sequences were then denoised using the DADA2 error filtering algorithm. Forward and reverse reads for Anserini/Cygnini primers were merged with a minimum overlap of 20 bp and a maximum of one accepted error in the overlap region, and putative chimeras were removed. For Anatini/Aythyini primers, because of the small size of the amplicon (200 bp and 179 bp respectively), only forward reads were used for subsequent analysis. The resulting dataset contains amplicon sequence variants (ASVs) and the associated read counts for each sample. Taxonomic assignments for the resulting amplicon sequence variants (ASVs) were obtained using the BLASTn algorithm [54] and the nucleotide and taxonomy (nt/taxdb) databases from NCBI [55,56]. We retained the top five target sequence matches for each ASV and assigned species-level taxonomy to ASVs that matched a single species with a sequence identity of ≥98%. If multiple species matched the query sequence equally well, ASVs were assigned the lowest common taxonomic rank (genus or family) among the target sequences with equal percent identity. ASVs that could not be classified to the family level were excluded from further analysis. ASVs with taxonomic assignments were filtered by removing ASVs with read counts fewer than the average number of non-zero reads summed across the DNA extraction and PCR negative controls. To account for variation in sequencing depth among samples, read counts were standardized by proportionally scaling each sample to the minimum sequencing depth prior to downstream metabarcoding analyses.

### Statistics

To account for abundances that differed across species by five orders of magnitude for both visual surveys and ASVs, we used log-log correlations to reflect the relationship between number of ASVs and the visual estimates of bird number, as previous quantitative eDNA metabarcoding studies have done [57,58].

Because waterfowl daily density is likely to exhibit short-term temporal autocorrelation [59] and because the rate of eDNA degradation can vary from hours to weeks depending on the ecosystem, target species, and eDNA capture method [60], we calculated the correlations between the number of ASVs and individuals, as well as between the number of birds/species (from eBird lists) on different dates, up to 5 days prior to each eDNA sampling event. We selected this period as a pragmatic approximation of “recent presence”, consistent with the timescales over which migrating waterfowl commonly use major stopover sites such as Montezuma NWR [61], and with the upper limit interval over which macro-organismal eDNA is often detectable in aquatic systems [62–64].

To visualize the temporal covariation of waterfowl and their associated eDNA reads during migration, we constructed a correlation-based network (CNA) including eDNA and visual count datasets. In the CNA, the nodes represent dates during the fall migration and the edges between them the significant, positive, log-log correlation coefficients (≥ 0.6) between visual surveys or between visual surveys and the standardized number of DNA reads obtain by combining the Anserini/Cygnini and Anatini/Aythyini datasets. We applied a correlation threshold of r ≥ 0.6 to retain only strong, positive associations, reducing network density and limiting the influence of weak or potentially spurious correlations that can arise from shared trends, compositional effects, or sampling noise. This threshold is commonly used in correlation-based ecological and microbial network analyses to improve interpretability and focus on biologically meaningful structure rather than marginal relationships (e.g., [65,66]).

To compare waterbird relative abundance estimates based on visual surveys with those based on eDNA read counts we focused on sampling dates with at least three independent complete eBird checklists (September 24th, October 15th, November 7th, and November 29th). For each of these dates, we compared the log-log correlation coefficient between our visual observations and eDNA counts to the log-log correlation coefficient between our visual observations and eBird data. All statistical analyses were performed with R.4.0.4 [67] and igraph.

## Results

### Evaluation of avian metabarcoding primers on eDNA samples

The pilot experiments at Sapsucker Woods Pond and the two Huyck Preserve waterbodies revealed a high level of cross-amplification of non-targeted taxa for all previously published avian metabarcoding primers. COI primers showed very little specificity, amplifying mostly bacteria (72% of reads) and fishes (20% of reads) (S4 Fig). Similarly, CytB primers amplified mostly other taxonomic groups (fish 54% of reads; mammals 44% (S4 Fig). 12S and ND2 primers amplified mostly avian species (86% and 78% of reads respectively), but the majority of ASVs were represented by only one waterfowl species (Canada goose) that was the predominant species in the two locations during the field sampling (S4 Fig). 12S and ND2 also cross-amplified teleosts (12S: 8% of reads; ND2: 20% of reads), potentially compromising the ability to detect waterfowl species in water bodies where fish species are abundant.

### In-silico and experimental evaluation of waterfowl-targeted primers

To avoid fish cross-amplification and increase waterfowl detection, we designed three pairs of primers, each of which targeted groups that corresponded to Anatidae tribes (Table 1).

In silico-PCR simulated against all vertebrate sequences available on the nt NCBI repository revealed that the targeted primers could amplify all ND2 waterfowl sequences available, encompassing 76% (132/174) of the species recognized by the IOC World Bird List International Ornithological Committee [68]. None of the targeted primers are waterfowl-specific when considering a maximum of two mismatches between each primer and the template. However, within these parameters the newly designed primers exclusively amplify bird species (S6 Fig). The experimental evaluation in two water bodies with contrasting waterfowl species diversity indicated high sensitivity and accuracy. At the Huyck Preserve, where visual surveys indicated low abundance and diversity of waterfowl (Canada goose, n = 406; Wood duck, n = 56; hooded merganser, n = 5; mallard, n = 11; American black duck, n = 1), we generated approximately 4 million high-quality reads, identifying 46 ASVs corresponding to four of the five observed species, with the Hooded Merganser being the only one not detected. Additionally, we identified DNA from three species that were not visually observed: American Wigeon, Northern Pintail, and Redhead. At Montezuma NWR, where 25 waterfowl species were reported during our sampling period (Table 2), the eDNA dataset comprised over 11 M high-quality sequence reads (~650,000 reads/sample) corresponding to 322 waterfowl ASVs. eDNA successfully detected all waterfowl species present in Montezuma NWR, whose abundance spanned 5 orders of magnitude (from less than 10 to over 5,000 individuals). While most of the ASVs could be undoubtedly assigned to a single species, the targeted ND2 fragments could not be distinguished three species pairs: mallard/American black duck, tundra swan/trumpeter swan, and greater/lesser scaup. Accordingly, both visual and ASV counts for these species pairs were pooled for subsequent analyses.

**Table 2:** Waterfowl species detected by our ground visual surveys, eBird checklists, and targeted metabarcoding (eDNA) during field sampling in 2020.

	Scientific name	Focal survey	eBird	eDNA
American Wigeon	*Mareca americana*			
Blue-winged Teal	*Spatula discors*			
Bufflehead	*Bucephala albeola*			
Cackling Goose	*Branta hutchinsii*			
Canada Goose	*Branta canadensis*			
Canvasback	*Aythya valisineria*			
Common Merganser	*Mergus merganser*			
Eurasian Wigeon	*Mareca penelope*			
Gadwall	*Mareca strepera*			
Greater/Lesser Scaup	*Aythya marila/Aythya affinis*			
Green-winged Teal	*Anas crecca*			
Hooded Merganser	*Lophodytes cucullatus*			
Mallard/American Black Duck	*Anas platyrhynchos/* *Anas rubripes*			
Northern Pintail	*Anas acuta*			
Northern Shoveler	*Spatula clypeata*			
Redhead	*Aythya americana*			
Ring-necked Duck	*Aythya collaris*			
Ross’s Goose	*Anser rossii*			
Ruddy Duck	*Oxyura jamaicensis*			
Snow Goose	*Anser caerulescens*			
Trumpeter/Tundra Swan	*Cygnus buccinator/Cygnus columbianus*			
Wood Duck	*Aix sponsa*			

### Temporal variation in the abundance of waterfowl species

**Fig 2:**
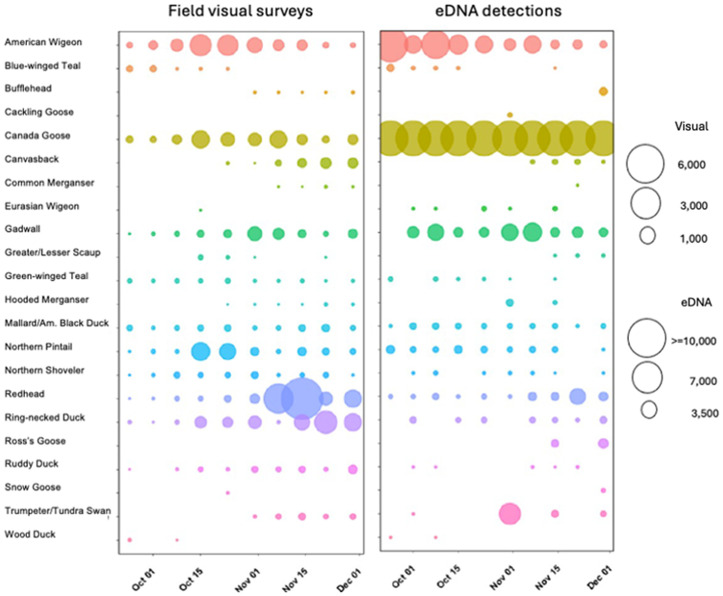
Temporal variation of visual surveys and eDNA standardized detections during waterfowl fall migration at Montezuma NWR.

Overall, there was a weak correlation between the standardized number of ASVs and the total abundance of birds, as determined by the number of individuals observed during the visual survey on the day of sampling (Fig 2). Only 8 out of 25 species (including blue-winged teal, bufflehead, canvasback, green-winged teal, and wood duck) showed a statistically significant correlation (P_one-tail_> 0.05). For example, Canada goose ASVs were consistently overrepresented, while ring-necked duck reads were underrepresented. Although both species exhibited strong seasonal variation in the number of individuals, there was little seasonal variation in their eDNA read counts. Thus, the observed variation in the standardized number of ASVs did not accurately reflect the temporal fluctuations in the absolute abundance of waterfowl species observed during fall migration.

**Fig 3:**
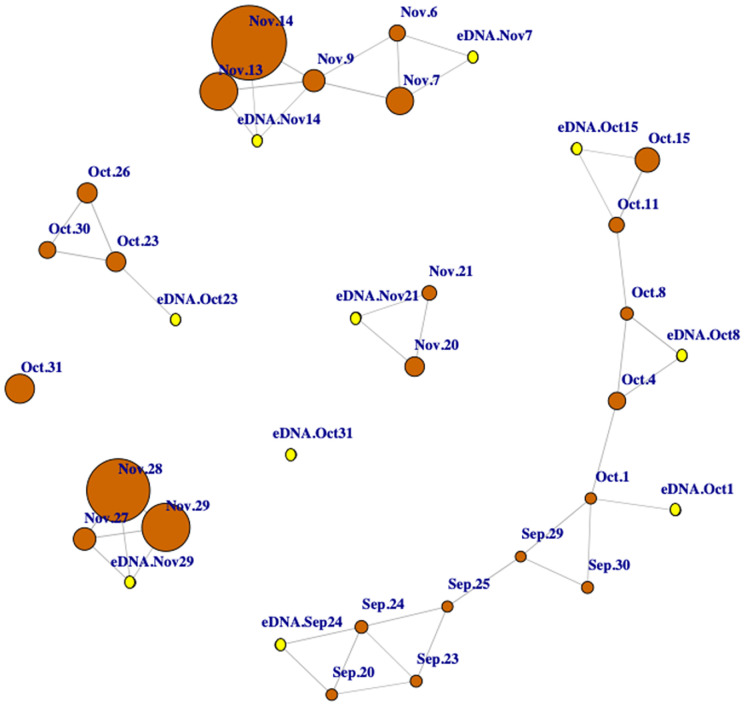
Correlation-based network of the standardized ASVs and the bird visual surveys. The nodes represent the dates during the waterfowl fall migration and the edges between them represent the significant, positive, and log-log correlation coefficients (≥ 0.6) between ground visual surveys (orange nodes) or between visual surveys and the standardized number of ASVs (pink nodes) obtain by combining the Anserini/Cygnini and Anatini/Aythyini and datasets.

In contrast, the standardized ASV counts showed significant correlations with relative abundance (Fig 3). A correlational network analysis of significant positive log-log relationships between ground visual surveys and standardized ASVs revealed a highly dynamic and variable system. Importantly, eDNA data captured relative waterfowl abundance not only on the day of sampling, but also correlated well with visual surveys conducted up to five days prior (Fig 3). The exception was October 31st, when the relative abundance of species in eDNA did not show any significant correlation with the relative abundance based on individual ground visual surveys (Fig 3).

**Fig 4:**
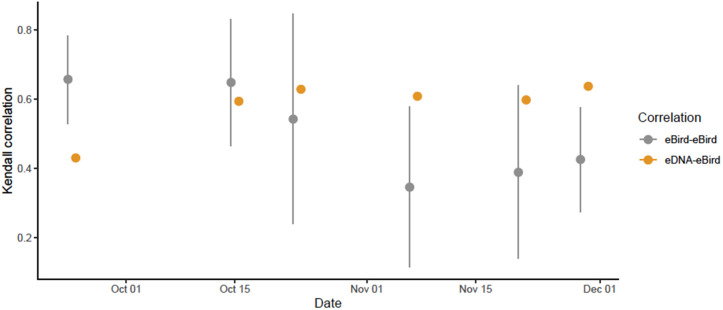
Abundance correlations between visual an eDNA based surveys at each sampling event. Gray: Mean and 95% confidence intervals of Kendall correlation coefficients between the number of birds present as determined by eBird checklists from different observers including our own visual survey. Orange: Kendall correlation coefficients between the standardized number of ASVs and number of birds present. We did not include the dates Oct1, Oct8, Oct31 and Nov 14 in this analysis because there were fewer than three checklists available for correlation.

At any given sampling date, the relative abundance of ASVs showed a positive correlation (average τ ≥ 0.62) with the relative abundance of species present in the area. On 5 out of the 6 sampling dates for which at least three independent ground visual surveys (eBird) were available, the strength of the eDNA-visual correlation exceeded that of the correlation between ground visual surveys (Fig 4).

## Discussion

Our study adds to the growing body of research demonstrating the usefulness of eDNA approaches for monitoring bird species, and extends this work by showing how eDNA can be applied to track species turnover and community dynamics during migration. While most previous avian eDNA studies have focused on the detection of specific target species, such as the Gouldian Finch [69], Black Rail [70], Ridgway’s Rail [71], and various wading birds [72,73], our study employs targeted metabarcoding, a technique inherently designed to characterize entire communities. By leveraging repeated sampling through a migration period, we demonstrate that eDNA metabarcoding can resolve temporal changes in community composition, rather than static presence alone. This work complements recent applications of metabarcoding to assess bird community composition across structurally complex habitats, including bush ecosystems [74], rice-based agricultural landscapes [75], and managed wetland systems supporting waterbird assemblages such as geese and swans [31,32,76], and highlights the potential of eDNA for dynamic biodiversity monitoring in systems where community composition is inherently transient.

By employing a suite of primers specifically designed to target Anatidae tribes, with reduced cross-amplification to non-target vertebrates relative to existing avian primers, we detected both common and rare waterfowl species during fall migration in an important bird conservation area, highlighting the value of eDNA metabarcoding for community-level presence–absence detection in birds. Notably, eDNA detections included all waterfowl species recorded during visual surveys, as well as Ross’s goose, an uncommon and difficult-to-identify species that often blends into mixed flocks with the more abundant snow goose.

Presence data are an important component of ecological inference, and can be used to determine migration time, stopover use, turnover, and habitat suitability [77,78]. Our results show that eDNA accurately determines the species composition of waterfowl communities and can, therefore, be used to capture broad-scale community dynamics, turnover, and temporal patterns. In some circumstances, eDNA signals vary substantially over short distances due to localized shedding, retention, and environmental heterogeneity. However our hydrologically integrative approach successfully aggregated eDNA signals over space (e.g., [79–81]: the eDNA samples collected at the outflow of the Main Pool at Montezuma NWR obtained a pooled, system-level assessment of species presence.

Our recent work on eDNA transport in Cayuga Lake, has shown that flow pathways, residence time, and vertical mixing strongly regulate eDNA signal strength, and that eDNA concentrations reflect the coupling of production with physical transport processes rather than abundance alone [82]. In impounded wetland systems such as Montezuma NWR, this complexity is likely to be further amplified by emergent and submerged vegetation, which can trap, retain, or locally concentrate eDNA [44,83,84]. Therefore, our quantitative inferences should not be generalized to study designs that employ spatially replicated sampling. For most waterfowl species in our study, the number of ASVs was a poor proxy of the total number birds present. Such a mismatch between ASV counts and the number of individuals present is often reported in metabarcoding studies and has been often highlighted as an intrinsic limitation of metabarcoding studies. In contrast, as in our study, the proportion of ASVs attributed to different waterfowl species reliably reflected their relative abundance, and the eDNA signal captured the seasonal shifts in the relative abundance of functional groups, with dabbling ducks more prevalent during early to mid-fall migration and diving ducks becoming more abundant later in the season. The ability of eDNA metabarcoding results to detect such shifts is important because, outside a migratory context, similar changes in community composition are widely used as early warning indicators of habitat degradation, climate-driven range shifts, and biodiversity loss [85–87]. Even in relatively resilient guilds such as waterfowl [9], changes in the balance between dabbling and diving ducks can signal alterations in vegetation structure or water depth, providing actionable information for adaptive wetland management [88].

The correlation coefficients between standardized ASV counts and visual surveys were similar in magnitude to the correlation coefficients obtained between different visual surveys. Visual surveys, including eBird, are subject to observer effort and skill, species detectability, and reporting biases, and therefore uncertainty. Consequently, the agreement between eDNA and eBird likely reflects shared sensitivity to broad temporal dynamics in migration phenology rather than a proof of methodological superiority or a direct validation of abundance estimates.

Altogether, our results demonstrate that eDNA-targeted metabarcoding captures ecologically meaningful signals of relative waterfowl abundance and temporal community dynamics. While this approach is not yet a replacement for traditional monitoring methods, it provides a powerful complementary tool for informing adaptive management, particularly in dynamic wetland ecosystems where environmental pressures, rapid change, and logistical constraints challenge conventional survey approaches.

## Supporting information

S1 Fig eDNA sampling site at the Cornell Lab of Ornithology, Ithaca, NY, sampling site SWP1. (TIF)

S2 Fig eDNA sampling sites at the Huyck Preserve, Rensselaerville, NY. Lake Lincoln Pond (P1 and P2) and Lake Myosotis (L1 – L3).Ten Mile Creek (C1), which links the two waterbodies. (TIF)

S3 Fig Workflow of the pilot experiments to conduct the initial assessment of both general and targeted bird metabarcoding primers (the Cornell Lab of Ornithology, Phase I, and the Huyck Preserve, Phase II). (TIF)

S4 Fig Percentage of sequence reads per organism group for each genetic marker tested to amplify bird DNA in environmental samples collected at Sapsucker Woods Pond and Huyck Preserve. (TIF)

S5 Fig Phylogenetic tree of waterfowl species selected in this study to design waterfowl ND2 primers. We used the Neighbor-Joining tree-built method and Tamura-Nei genetic distance model in Geneious Prime 2020.2.1. Colors represent different Tribes: red (Anserini and Cygnini), green (Anatini, Cairinini, and Oxyura), and orange (Aythyini and Mergini).
(TIF)

S6 Fig Graphical summary of the taxonomic families amplified by waterfowl ND2 primers. (A) Anatini and Cairinini ND2 Primers, (B) Aythyini and Mergini ND2 Primers, and (C) Anserini and Cygnini ND2 Primers.
(PDF)

S1 Table Mitochondrial genes and multiple primers tested in the pilot experiments to amplify bird DNA from eDNA water samples. Different colors in the table represent the primer pair. Mibird-U-F1, F2, F3, and F4 were used in pair with Mibird-U-R.
(PDF)

S2 Table Insert length and amplicon size of each genetic marker tested in this study. (PDF)

S1 File Supporting information. (DOCX)
