# Supplementary figures and images for: Environmental DNA monitoring of waterfowl reveals community changes during migration
Source: PLoS One. 2026 Apr 28;21(4):e0337508. doi: 10.1371/journal.pone.0337508 (PMC13123992; doi:10.1371/journal.pone.0337508)

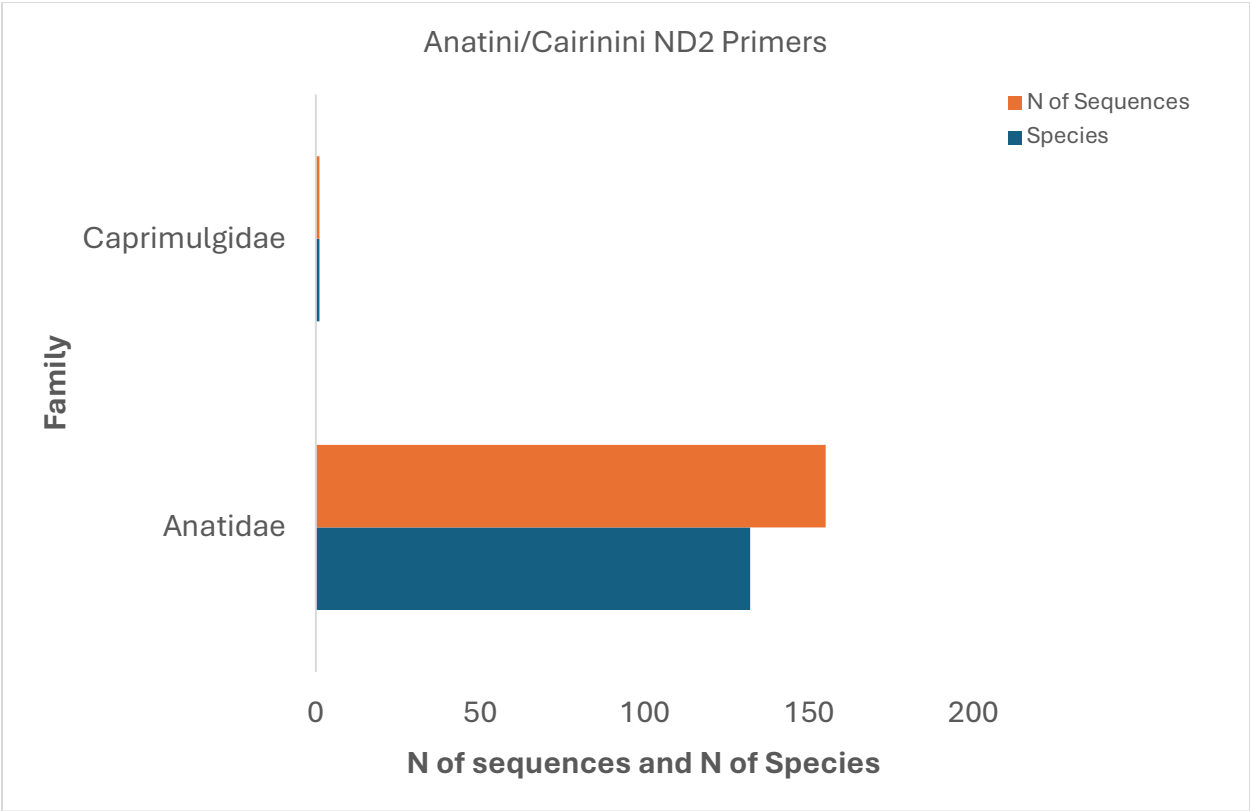

(A)

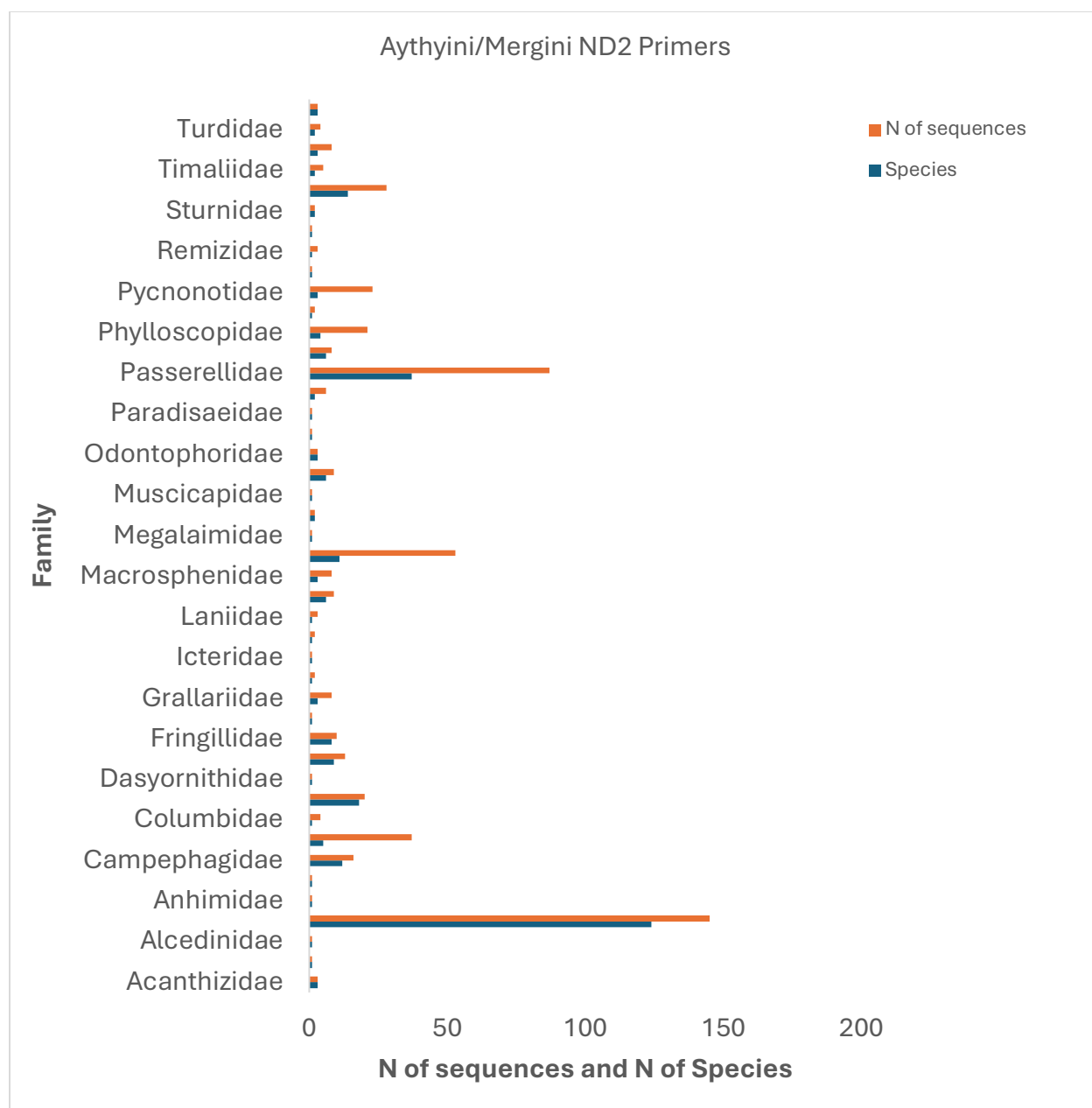

(B)

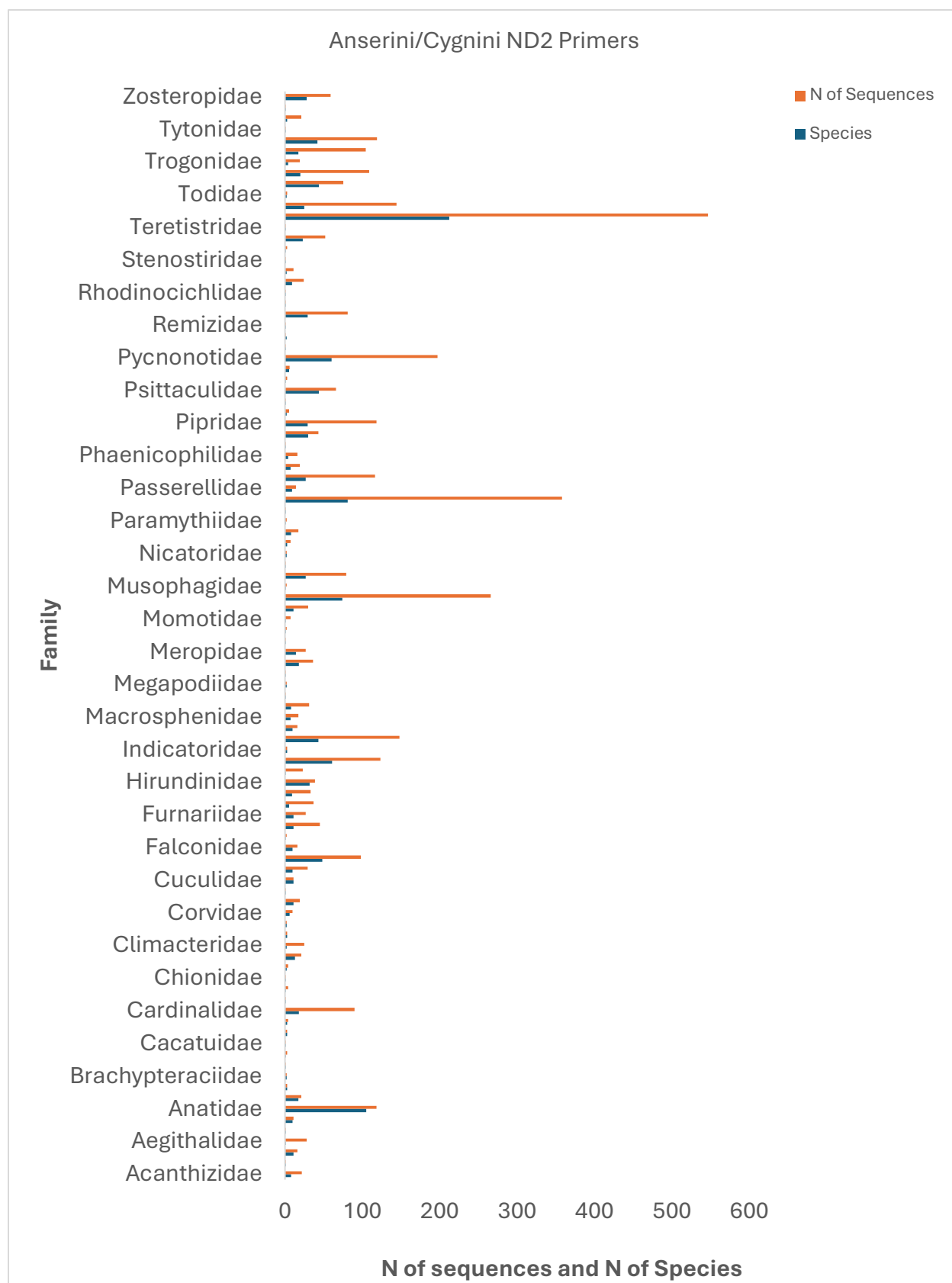

(C)

Supplement: S6 Fig — (A) Anatini and Cairinini ND2 Primers, (B) Aythyini and Mergini ND2 Primers, and (C) Anserini and Cygnini ND2 Primers. (PDF) [file pone.0337508.s006.pdf]
