# Supplementary material for: Environmental DNA monitoring of waterfowl reveals community changes during migration
Source: PLoS One. 2026 Apr 28;21(4):e0337508. doi: 10.1371/journal.pone.0337508 (PMC13123992; doi:10.1371/journal.pone.0337508)
Supplement: S1 Table — Different colors in the table represent the primer pair. Mibird-U-F1, F2, F3, and F4 were used in pair with Mibird-U-R. (PDF) [file pone.0337508.s007.pdf]

Gene	Primer	Direction	Primer sequence (5' - 3')	Reference
COI	BirdF1	Forward	TTCTCCAACCACAAAGACATTGGCAC	Kerr *et al*., 2007
COI	BirdR1	Reverse	ACGTGGGAGATAATTCCAAATCCTG	Kerr *et al* l., 2007
COI	FalcoFA	Forward	TCAACAAACCACAAAGACATCGGCAC	Kerr *et al*., 2007
COI	BirdR1	Reverse	ACGTGGGAGATAATTCCAAATCCTG	Kerr *et al*., 2007
12S rRNA	MiBird-U-F	Forward	GGGTTGGTAAATCTTGTGCCAGC	Ushio *et al*., 2018
12S rRNA	MiBird-U-R	Reverse	CATAGTGGGGTATCTAATCCCAGTTTG	Ushio *et al*., 2018
12S rRNA	Aves_12Sa	Forward	GATTAGATACCCCACTATGC	Epp *et al*., 2012
12S rRNA	Aves_12Sc	Reverse	GTTTTAAGCGTTTGTGCTCG	Epp *et al*., 2012
12S rRNA	MiBird-U-F1	Forward	TAAATCYTGTGCCAGCCACCGCGG	Modified from Ushio *et al*., 2018
12S rRNA	MiBird-U-F2	Forward	TAAATCTTGTGCCAGCTACCGCGG	Modified from Ushio *et al*., 2018
12S rRNA	MiBird-U-F3	Forward	TAAATCTTGTGCCAGCCGCCGCGG	Modified from Ushio *et al*., 2018
12S rRNA	MiBird-U-F4	Forward	TAGATCTTGTGCCAGCCACCGTGG	Modified from Ushio *et al*., 2018
ND2	ND2 L5216	Forward	GGCCCATACCCCGRAAATG	Sorenson 2003
ND2	ND2 H5766	Reverse	RGAKGAGAARGCYAGGATYTTKCG	Sorenson 2003
Cytb	Cytb L14816	Forward	CCATCCAACATCTCAGCATGATGAAA	Awad *et al*., 2015, Kocher *et al*., 1989
Cytb	Cytb H15173	Reverse	CCCCTCAGAATGATATTTGTCCTC	Awad *et al*., 2015, Kocher *et al*., 1989
COI	AWCF1	Forward	CGCYTWAACAYTCYGCCATCTTACC	Patel *et al.,* 2010
COI	AWCintR2	Reverse	ATGTTGTTTATGAGTGGGAATGCTATG	Patel *et al*., 2010
