# Supplementary material for: Environmental DNA monitoring of waterfowl reveals community changes during migration
Source: PLoS One. 2026 Apr 28;21(4):e0337508. doi: 10.1371/journal.pone.0337508 (PMC13123992; doi:10.1371/journal.pone.0337508)
Supplement: S2 Table — (PDF) [file pone.0337508.s008.pdf]

Gene	Insert length	Amplicon size	Used for eDNA	eDNA Bird detection	Reference
**COI**	658	710	No	-	Kerr et al., 2007
**COI internal primer**	328	380	No	-	Patel et al., 2010
**12S rRNA MiBird**	171	221	Yes	Yes, but included amplification of non-target organism groups (fish and mammals)	Ushio et al., 2018
**ND2**	550	593	No	-	Sorenson 2003
**Cytb**	357	407	No	-	Awad et al., 2015
**12S rRNA Aves**	50	90	Yes	Yes, but low taxonomic resolution	Epp et al., 2012
**12S rRNA MiBird modified**	171	222	-	-	Modified from Ushio et al., 2018
