# Supplementary material for: Environmental DNA monitoring of waterfowl reveals community changes during migration
Source: PLoS One. 2026 Apr 28;21(4):e0337508. doi: 10.1371/journal.pone.0337508 (PMC13123992; doi:10.1371/journal.pone.0337508)
Supplement: S1 File — (DOCX) [file pone.0337508.s009.docx]

**Supporting information**

Our pilot study included the following steps: (1) selection of genetic markers and testing of multiple primer pairs to amplify avian DNA using a mock community, (2) field sampling design, (3) molecular analyses, (4) High-throughput sequencing, (5) bioinformatic pipeline analysis, and (6) identification of avian species. An avian mock community was designed using five avian species (American Wigeon, Osprey, Vegetarian Finch, Northern Flicker, Sanderling). The mock community was designed using an equal molar DNA sample with a concentration of 10 ng/μL for each avian species. The mock community was used to test the efficiency of the primer set to amplify avian DNA of different avian species. Our goal was to amplify small fragments of avian mitochondrial DNA (mtDNA). Four genetic markers were selected based on previous studies and the number of sequences present in the GenBank database (S2 Table). Eleven primer pairs were tested with each bird species and with the mock community (S1 Table).

At the Sapsucker Woods we collected two 250 mL samples and one negative control in one site in August 2019: (S1 Fig). At the Huyck Preserve we sampled two sites at a small pond, Lincoln Pond with nine hectares, and three sites at a small lake, Lake Myosotis with around 30 hectares, during the fall bird migration in two periods (early October and later October 2020) (S2 Fig). Lake Myosotis and Lincoln Pond have hosted 25 different species of waterbirds (https://www.huyckpreserve.org/newsletter-resources.html). A total of five sites were sampled and five replicates (250 mL) and one negative control were collected per site.

**S1 Table.** **Mitochondrial genes and multiple primers tested in the pilot experiments to amplify bird DNA from eDNA water samples.** Different colors in the table represent the primer pair. Mibird-U-F1, F2, F3, and F4 were used in pair with Mibird-U-R.

| **Gene** | **Primer** | **Direction** | **Primer sequence (5' - 3')** | **Reference** |
| --- | --- | --- | --- | --- |
| COI | BirdF1 | Forward | TTCTCCAACCACAAAGACATTGGCAC | Kerr *et al*., 2007 |
| COI | BirdR1 | Reverse | ACGTGGGAGATAATTCCAAATCCTG | Kerr *et al* l., 2007 |
| COI | FalcoFA | Forward | TCAACAAACCACAAAGACATCGGCAC | Kerr *et al*., 2007 |
| COI | BirdR1 | Reverse | ACGTGGGAGATAATTCCAAATCCTG | Kerr *et al*., 2007 |
| 12S rRNA | MiBird-U-F | Forward | GGGTTGGTAAATCTTGTGCCAGC | Ushio *et al*., 2018 |
| 12S rRNA | MiBird-U-R | Reverse | CATAGTGGGGTATCTAATCCCAGTTTG | Ushio *et al*., 2018 |
| 12S rRNA | Aves_12Sa | Forward | GATTAGATACCCCACTATGC | Epp *et al*., 2012 |
| 12S rRNA | Aves_12Sc | Reverse | GTTTTAAGCGTTTGTGCTCG | Epp *et al*., 2012 |
| 12S rRNA | MiBird-U-F1 | Forward | TAAATCYTGTGCCAGCCACCGCGG | Modified from Ushio *et al*., 2018 |
| 12S rRNA | MiBird-U-F2 | Forward | TAAATCTTGTGCCAGCTACCGCGG | Modified from Ushio *et al*., 2018 |
| 12S rRNA | MiBird-U-F3 | Forward | TAAATCTTGTGCCAGCCGCCGCGG | Modified from Ushio *et al*., 2018 |
| 12S rRNA | MiBird-U-F4 | Forward | TAGATCTTGTGCCAGCCACCGTGG | Modified from Ushio *et al*., 2018 |
| ND2 | ND2 L5216 | Forward | GGCCCATACCCCGRAAATG | Sorenson 2003 |
| ND2 | ND2 H5766 | Reverse | RGAKGAGAARGCYAGGATYTTKCG | Sorenson 2003 |
| Cytb | Cytb L14816 | Forward | CCATCCAACATCTCAGCATGATGAAA | Awad *et al.*, 2015, Kocher *et al*., 1989 |
| Cytb | Cytb H15173 | Reverse | CCCCTCAGAATGATATTTGTCCTC | Awad *et al.*, 2015, Kocher *et al*., 1989 |
| COI | AWCF1 | Forward | CGCYTWAACAYTCYGCCATCTTACC | Patel *et al.,* 2010 |
| COI | AWCintR2 | Reverse | ATGTTGTTTATGAGTGGGAATGCTATG | Patel *et al*., 2010 |

**S2 Table. Insert length and amplicon size of each genetic marker tested in this study.**

| **Gene** | **Insert length** | **Amplicon size** | **Used for eDNA** | **eDNA Bird detection** | **Reference** |
| --- | --- | --- | --- | --- | --- |
| **COI** | 658 | 710 | No | - | Kerr et al., 2007 |
| **COI internal primer** | 328 | 380 | No | - | Patel et al., 2010 |
| **12S rRNA**  **MiBird** | 171 | 221 | Yes | Yes, but included amplification of non-target organism groups (fish and mammals) | Ushio et al., 2018 |
| **ND2** | 550 | 593 | No | - | Sorenson 2003 |
| **Cytb** | 357 | 407 | No | - | Awad et al., 2015 |
| **12S rRNA**  **Aves** | 50 | 90 | Yes | Yes, but low taxonomic resolution | Epp et al., 2012 |
| **12S rRNA**  **MiBird modified** | 171 | 222 | - | - | Modified from Ushio et al., 2018 |

**
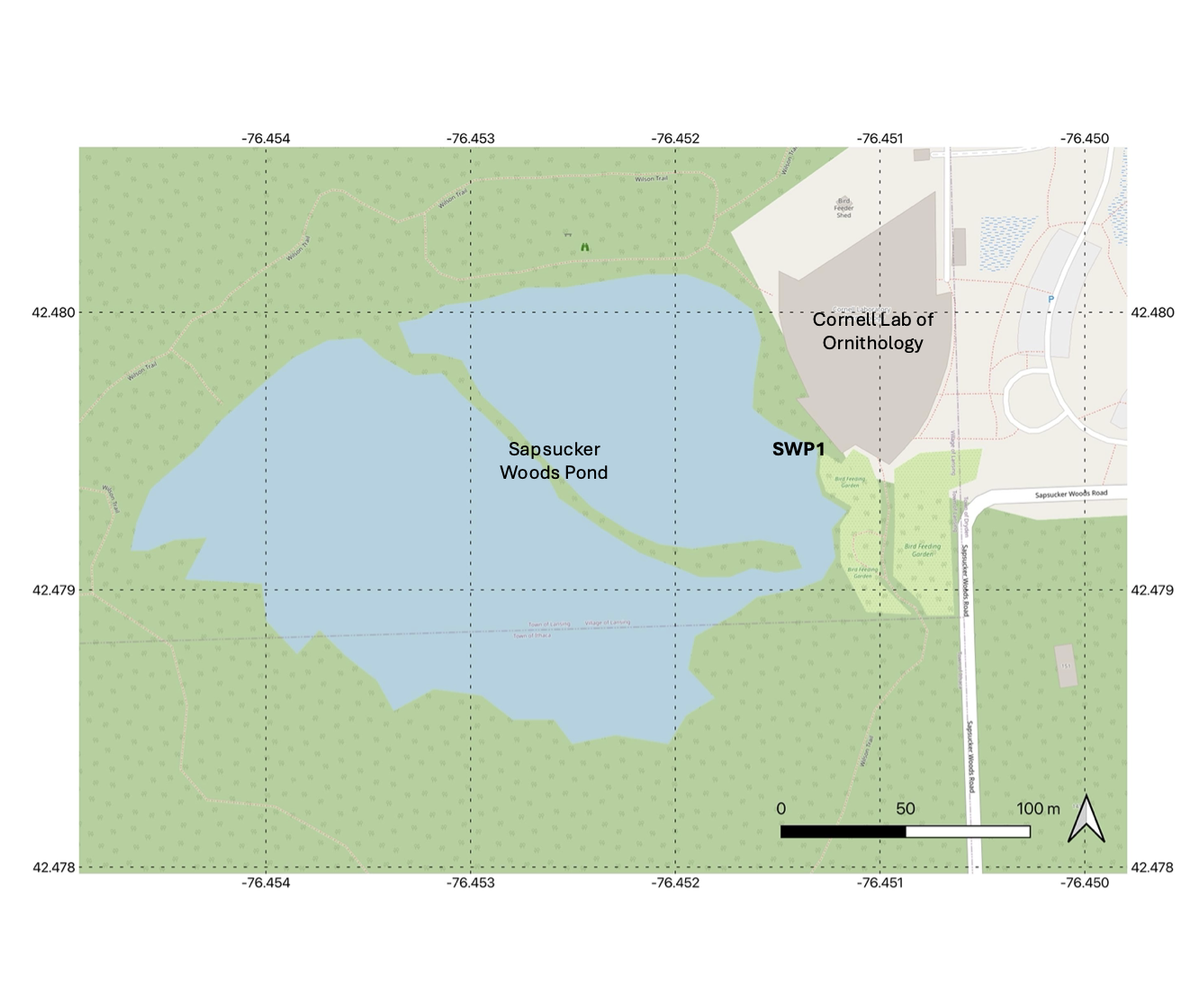
**

**S1 Fig. eDNA sampling site at the Cornell Lab of Ornithology, Ithaca, NY, sampling site SWP1.**

**
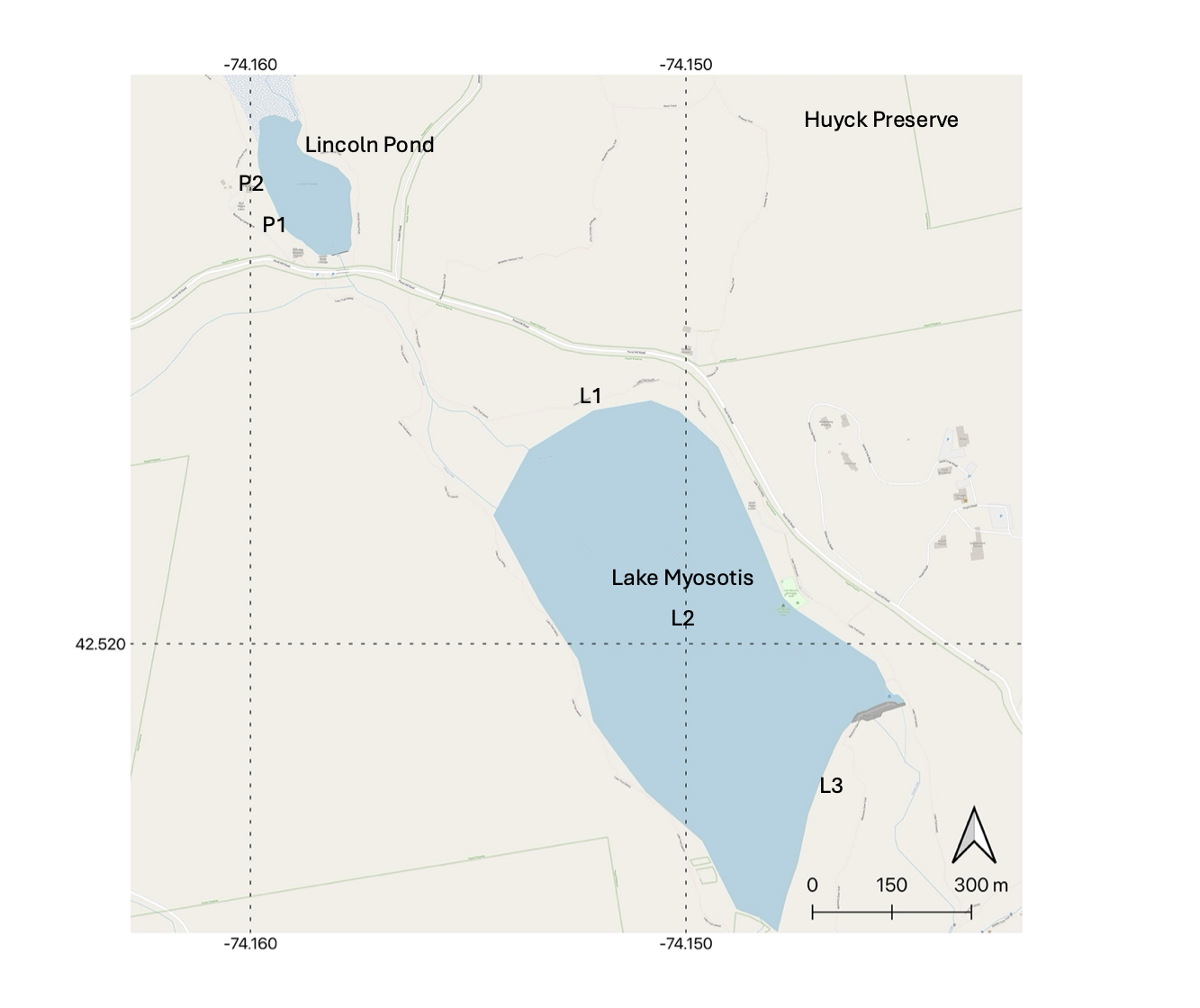
**

**S2 Fig. eDNA sampling sites at the Huyck Preserve, Rensselaerville, NY. Lake Lincoln Pond (P1 and P2) and Lake Myosotis (L1 – L3).Ten Mile Creek (C1), which links the two waterbodies.**

**
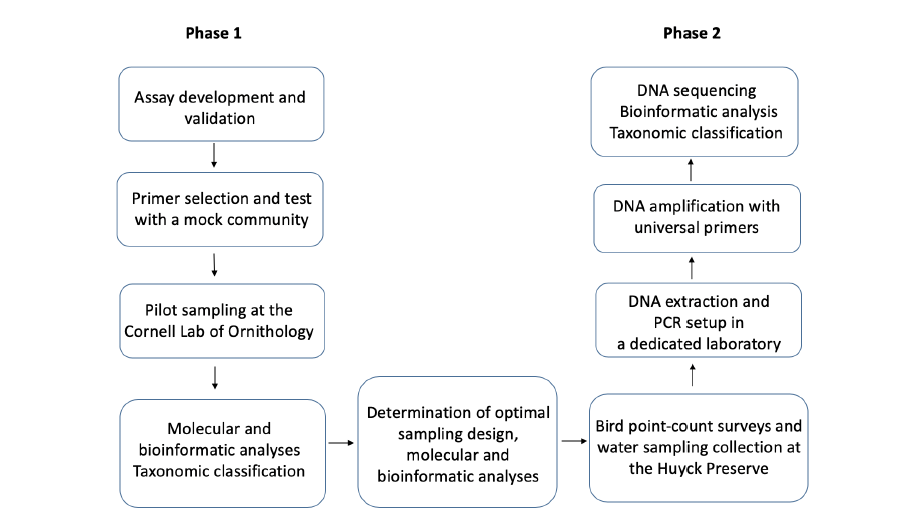
**

**S3 Fig. Workflow of the pilot experiments to conduct the initial assessment of both general and targeted bird metabarcoding primers (the Cornell Lab of Ornithology, Phase I, and the Huyck Preserve, Phase II).**


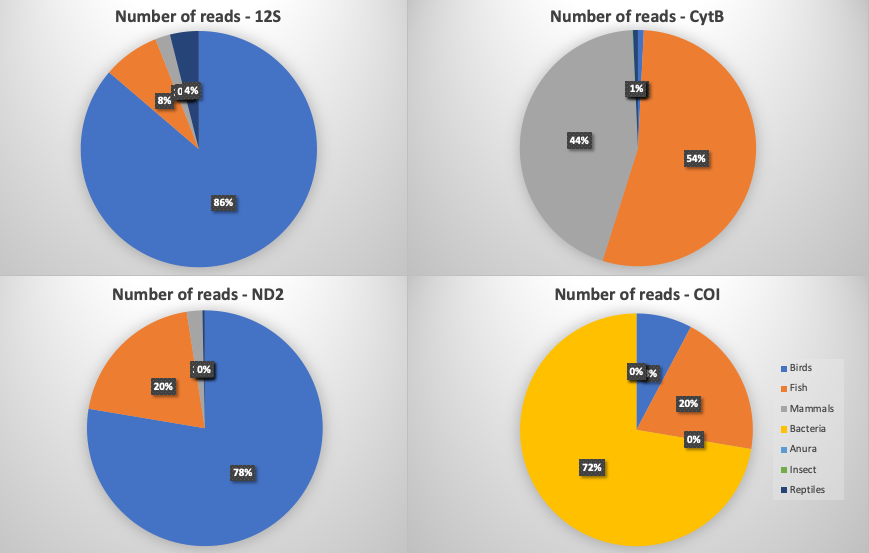


**S4 Fig**. **Percentage of sequence reads per organism group for each genetic marker tested to amplify bird DNA in environmental samples collected at Sapsucker Woods Pond and Huyck Preserve.**

**S5 Fig**. **Phylogenetic tree of waterfowl species selected in this study to design waterfowl ND2 primers.** We used the Neighbor-Joining tree-built method and Tamura-Nei genetic distance model in Geneious Prime 2020.2.1. Colors represent different Tribes: red (Anserini and Cygnini), green (Anatini, Cairinini, and Oxyura), and orange (Aythyini and Mergini).

**(A)**

**(B)**

**(C)**

**S6 Fig**. **Graphical summary of the taxonomic families amplified by waterfowl ND2 primers.** (A) Anatini and Cairinini ND2 Primers, (B) Aythyini and Mergini ND2 Primers, and (C) Anserini and Cygnini ND2 Primers.

**PCR thermal cycling parameters for designed ND2 primers**

PCR cycling parameters for Anserini/Cygnini primers (197F / 524R) were initial denaturation at 95ºC for 1 min, followed by 5 cycles of denaturation at 94ºC for 30s, annealing at 56ºC for 45s, extension decreasing by 1ºC every cycle and incubate at 72ºC for 45s, followed by 35 cycles of extension incubating at 94ºC for 20 s, 54ºC for 30s and 72ºC for 20s, with a final extension at 72ºC for 5 min.

PCR cycling parameters for Anatini/Cairinini/Oxyura primers (419F / 659R) were initial denaturation at 95ºC for 1 min, followed by 11 cycles of denaturation at 94ºC for 30s, annealing at 58ºC for 45s, extension decreasing by 1ºC every cycle and incubate at 72ºC for 45s, followed by 34 cycles of extension incubating at 94ºC for 20 s, 55ºC for 30s and 72ºC for 20s, with a final extension at 72ºC for 7 min.

PCR cycling parameters for Aythyini/Mergini primers (220F / 436R) were initial denaturation at 95ºC for 1 min, followed by 10 cycles of denaturation at 94ºC for 30s, annealing at 56ºC for 45s, extension decreasing by 1ºC every cycle and incubate at 72ºC for 45s, followed by 35 cycles of extension incubating at 94ºC for 20 s, 52ºC for 30s and 72ºC for 20s, with a final extension at 72ºC for 7 min.
